# Health issues in a Bangalore slum: findings from a household survey using a mobile screening toolkit in Devarajeevanahalli

**DOI:** 10.1186/s12889-019-6756-7

**Published:** 2019-04-29

**Authors:** Carolin Elizabeth George, Gift Norman, Avanti Wadugodapitya, Shyam Vasudeva Rao, Shailendra Nalige, Varshapriya Radhakrishnan, Sapna Behar, Luc de Witte

**Affiliations:** 1 Division of Community Health and Family Medicine, Bangalore Baptist Hospital, Bellary Road, Hebbal, Bangalore, 560024 India; 2 Zuyd University of Applied Sciences, Nieuw Eyckholt 300, 6419 DJ Heerlen, The Netherlands; 3 E Health Enablers Innovations Pvt. Ltd, Binnamangala, Stage 1, Indiranagar, Bangalore, India; 4 Icarus Nova, No 7, Rogers Road, Richards Town, Bangalore, India; 5 Centre for Assistive Technology and Connected Healthcare, University of Sheffield, Portobello, Sheffield, UK

**Keywords:** Slum, Screening, Health problems, Technology

## Abstract

**Background:**

Slums are home to nearly one billion people in the world and are expanding at an exponential rate. Devarjeevanahalli is a large notified slum in Bangalore, South India which is characterised by poverty, overcrowding, hazardous living environment and social complexities. The poor living conditions not only affect the health of the people living there but also poses distinctive challenges to conducting health surveys. The purpose of this paper is to report the findings of a household survey that was done to study the health condition of people living in a slum.

**Methods:**

A community-based cross-sectional survey was designed to determine the prevalence of health conditions using a mobile screening toolkit-THULSI (Toolkit for Healthy Urban Life in Slums Initiative). Devarjeevanahalli slum was chosen purposively as it is fairly representative of any slum in a big city in India. Sample size was calculated as 1100 households and demographic parameters at the household level and parameters related to priority health conditions (hypertension, diabetes mellitus, anaemia and malnutrition) at the individual level were studied.

Six zones within the slum were purposively selected and all the contiguous households were selected. The last of the six zones was partially surveyed as the desired sample size was achieved.

**Results:**

A total of 1186 households were surveyed and 3693 people were screened. More than three fourth (70.4%) of the population were below poverty line. Only one third had a regular job and the average daily income was 5.3$ and 2.6$ in men and women respectively. The prevalence of hypertension (35.5%), diabetes (16.6%) and anaemia (70.9%) was high in the screened slum population. Most of the people (56.5% of hypertensives and 34.4% diabetics) were screened for the first time. Almost half of the children under the age of five years were stunted.

**Conclusions:**

Poor income security and huge burden of health issues were reported among adults and children in the household health screening in a large notified slum in South India. Most people were unaware of their disease condition prior to the screening. Relatively simple technological solutions enabled the local health team to screen the slum population despite many challenges.

**Electronic supplementary material:**

The online version of this article (10.1186/s12889-019-6756-7) contains supplementary material, which is available to authorized users.

## Background

The United Nations Human Settlements Programme (UN-Habitat) defines a slum as“a group of individuals that live under the same roof that lack one or more of the following conditions: access to improved water, access to improved sanitation, sufficient living space, durability of housing and secure tenure” [1].Urban slums constitute one of the most disadvantaged sections of society. Adverse weather conditions and lack of job opportunities push people in villages to migrate to towns and cities. Economic stagnation, failure of redistribution, market distortion in favour of extractive elites, lack of planning and corruption have been postulated to explain how slums form, grow and persist [2]. People from rural areas ultimately settle in clustered unhygienic settlements in cities that form slums. The slums thus formed are characterised by high population density, dilapidated structures, lack of safe water and sanitation, and heaps of garbage, which make the environment highly conducive to diseases [2].

Health is a major challenge in slums due to these neighbourhood effects [3]. There is low acceptance for prevention in the midst of other pressing challenges like food and shelter. Most people only realize the need for health when it is lost. They may seek healthcare when they are very ill, but most of them cannot afford existing medical services. In addition, the fluidity of the physical environment and lack of a postal address pose unique challenges in following up on individuals with morbidities.

Surveying slums is a challenging task, hence it is reported in the literature that little is known about the spectrum and burden of disease morbidity in urban slums [4]. Surveying slum populations is challenging due to a variety of reasons: illegal squatters could avoid surveys [5]; high incidence of crimes and alcoholism [1]; poor health literacy [6]; social distance between the surveyors and surveyed [7]; difficulty in finding skilled surveyors to measure diseases; non-availability of tests in the slums [1]. All the above can pose challenges to measuring health parameters in these populations. On most occasions, what is known about slum health depicts only the tip of the iceberg; assumptions are usually based on clinic or hospital data [4].

Having an overall picture of the demography and health condition of slum population is essential to design innovations that address the unique challenges faced by them, and to develop services that meet their needs. Hence the purpose of the present survey was to describe the overall health condition of the slum population, which has the potential to add to the scarce literature available about health of the people in slums.

A local hospital - the Bangalore Baptist Hospital (BBH) offers primary medical care in this slum at present. However, BBH seeks to reach more people in need of healthcare and align its service with the needs of the population more optimally. Together with local companies and two academic institutions, BBH developed a screening toolkit [8] that enabled door to door screening of the priority diseases (based on prevalence, seriousness, feasibility of screening and potential benefits of early detection) in the slum population. The objective of the survey was to screen and document priority health conditions of slum inhabitants using the toolkit developed**.** This paper presents the results of a survey in which people living in 1186 households in the slum were screened using this toolkit.

## Methods

### Study design and setting

There are 2397 notified slums in Karnataka State, of which 387 are situated in Bangalore City alone. It is estimated that the slums within the State are home to a population of approximately 4.5 million - 22.56% of the State’s urban population. Devarajeevanahalli(DJ Halli) - one of the largest government notified slums in Bangalore, extending over 1.15 km with 420 huts and a ‘registered’ population of 2463was selected for the study [9]. The area is the urban field practice area of the Department of Community Health of the Bangalore Baptist Hospital. Contrary to the official statistics of around 2500 inhabitants, the population was found to be close to 50,000 (approximately 11,000 huts) based on community discussions and observation.

A community-based cross sectional study was designed to fulfil the objective.

Ethical approval for the study was obtained through the Institutional Review Board of Bangalore Baptist Hospital. Informed consent was taken in three stages. The local leaders were asked permission to conduct health survey in the first stage, the head of the household was asked permission to collect information from members of the household and then consent was taken on an individual basis to collect data as well as to collect blood if the individual fulfilled eligibility for invasive tests.

### Sample size

The study estimated two types of variables: demographic parameters at the household level and parameters related to priority health conditions at the individual level. Minimum sample size was estimated using the formula n = [Np(1-p)]/ [(d^2^/Z^2^1-α/2*(N-1) + p*(1-p)] where *Z* is the Z-score (*Z* is 1.96 for a 95% confidence level), where Np is the number of households in case of demographic variables and the population size slum in case of estimation of priority health conditions, *d* is the margin of error, *p* is the estimated proportion of an attribute (prevalence of hypertension, anemia etc) [10]. We have used a width of 6% instead of 10% (most often used across disciplines) to overestimate the number of samples (in case of missed households) [7]. For estimating demographic variables, we used a proportion of 0.5 which indicates the maximum variability of an attribute in a population. This is often used in determining a more conservative sample size. For estimating priority health conditions, we substituted *p* with prevalence of particular health condition from the literature. After calculating minimum sample size (261), it was decided to sample 10% of the slum households (1100) to get a conservative estimate on all the parameters studied considering missing households based on expert consultation. The detailed sample size calculation is explained in Table 1.

**Table 1:** Minimum sample size required for estimating demographic variables and priority health conditions

Parameter to be estimated	Sampling unit	Prevalence in other studies	Estimated minimum sample size *n* = [Np(1-p)]/ [(d^2^/Z^2^1-α/2*(N-1) + p*(1-p)] [10]	Values used for sample size calculation
Hypertension among adults	Adults 30 years or above	42% [11]	259	Population size 50,000, *p:* 42%, *d:* 6%, Confidence level 95%
Diabetes among adults	Adults 30 years or above	12% [12].	113	Population size 50,000, *p*: 12%, *d:* 6%, Confidence level 95%
Anaemia in women	Females 12 years and above	53% [13]	265	Population size 50,000, *p:* 53%, *d:* 6%, Confidence level 95%
Malnutrition in under 5 children	Children less than 5 years	50% [14]	248	Population size 5000, *p*: 50%, *d*: 6%, Confidence level 95%
Demographic parameters	Household	50% (to get the maximum sample size) [7]	261	Total number of households: 11000, *p*: 50%, *d*: 6%, Confidence level 95%

### Sampling and recruitment

DJ Halli is divided into two sub areas called as wards for administrative purposes with a total estimated population of 50,000 living in approximately 11,000 households. Each ward is arbitrarily divided into 12 zones based on community landmarks. The leaders selected one ward (BBH clinic is in this ward) and chose six zones (based on socio-economic condition) from that ward for survey purposes. They were Sujana Nagar, PunjappaLane, Thangamalai Nagar (together cluster A, mainly Hindus in relatively better socioeconomic condition, as opined by leaders), Indirapuram, Roshan Nagar, and Eidgarh (together cluster B, mostly Muslims, socioeconomically backward compared to cluster A, as opined by leaders).

Given that many households in DJ Halli do not have an established address system, a base map of its six zones showing the roads, lanes, nearest landmarks like mosques, churches, schools and well-known shops was developed. Lanes were numbered and houses were given a nomenclature to incorporate the area name and lane number. This number was written on the doors to aid in locating the houses. Pilot mapping of the first hundred houses showed that 43% of the houses were locked and unavailable for surveying. Based on this data, all the houses (2143) in the six zones were mapped for the survey.

Of the six zones selected, survey started in the zone farthest to BBH clinic. The first house in the beginning of a lane was selected and then contiguous households were recruited and surveyed till the end of the lane. Once all the houses in that zone was completed, the survey team moved to the next geographically adjacent zone. It was decided to stop the screening process when we achieved the planned sample size of 1100 households.

### Community preparation

Community meetings were held to explain the purpose of the survey. In these meetings, convenient dates and timings for the survey were discussed and recorded. Local leaders and politicians were met to gain support for the initiative.

### The screening toolkit

Field-based screening for priority health conditions carried out as part of the study, was done using the THULSI (Toolkit for Healthy Urban Life in Slums Initiative) toolkit. This mobile multifunctional and modular toolkit was developed through an international collaborative project implemented by Bangalore Baptist Hospital, the University of Sheffield, ZUYD University of Applied Sciences, e-Health enablers and Icarus Nova, based on the findings of an initial study to identify priority health issues in the area [8].

**Fig. 1:**
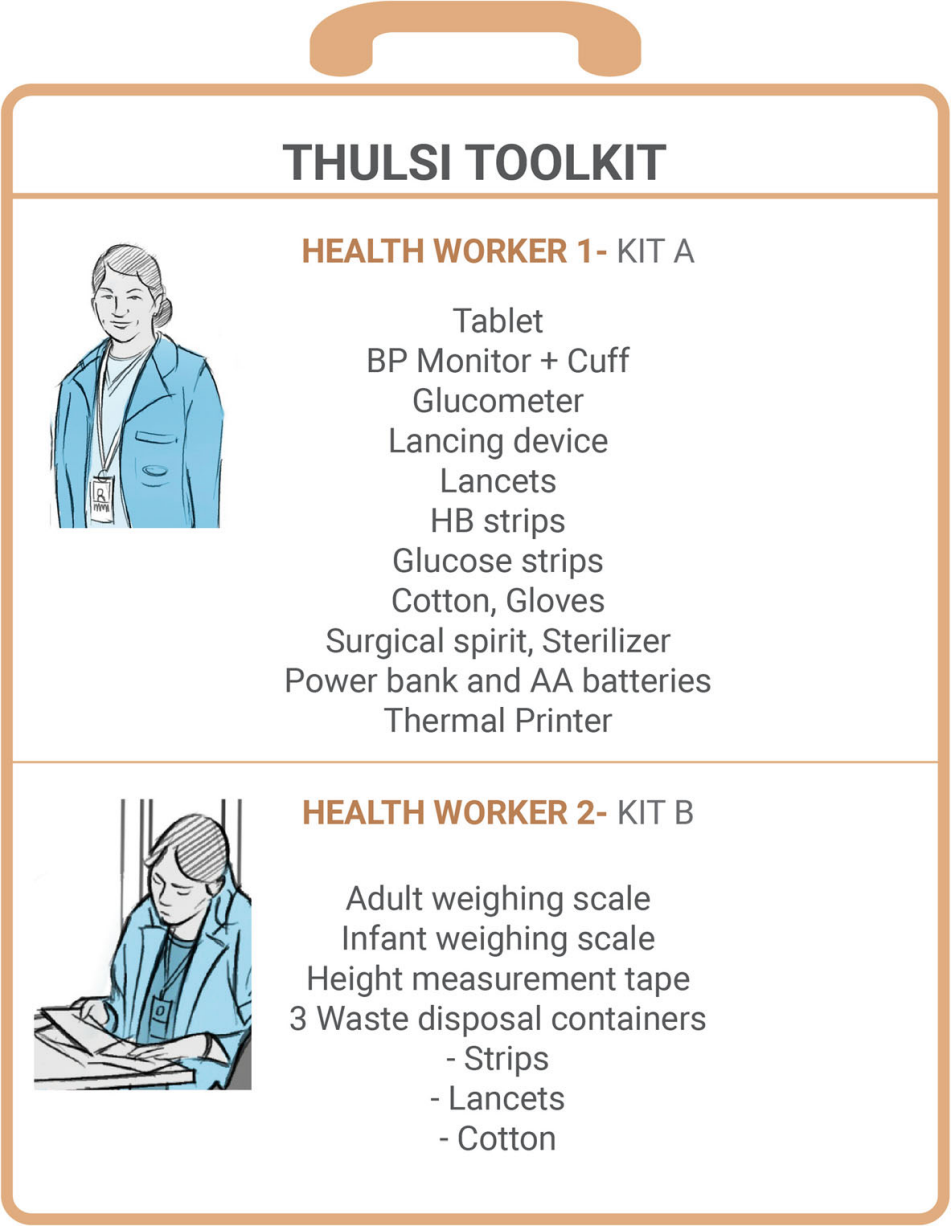
Description of THULSI with its components

THULSI consists of test devices and an android tablet, as well as a purpose-built software application. This application (App) is capable of documenting digitally key health parameters at the household, family and individual levels. The App was designed to help collect the demographic data as well as the health parameters in the simplest way possible. In order to minimize the errors in data entry during the survey, most of the fields were provided with a drop down menu and consistency checks. The app enabled the updating of data at all levels if the surveyor wanted to add details of a family member who was not available during the first screening. The App also enabled interfacing with a bluetooth enabled thermal printer to print the results of the health parameters being checked. The content and design of THULSI toolkit is presented in Fig. 1 and Additional file 1.

### Survey questionnaire and pilot testing

A questionnaire integrated into, and accessed via, the THULSI mobile software application on the mobile tablet, and complemented by the physical contents of THULSI, was used to carry out the survey. The survey questionnaire consisted of socio-demographic information, morbidity questions and recording of selected anthropometric measurements and health parameters. The information was collected in three levels 1) household (area, address, type of house, no. of rooms); 2) family (children going to school, ration card, health insurance, health seeking behaviour, morbidity, mortality in the last year);and 3) individual (age, sex, education, occupation, income, height, weight, BMI (Body Mass Index), Haemoglobin (Hb), blood pressure and random blood sugar measurement).

Prior to the commencement of data collection, the data collection teams were trained on how to use the toolkit and collect data for the study by the developers of the toolkit, including a week-long field-based mock data collection exercise.

### Data collection

Data collection was carried out by five teams, each consisting of four to five nursing students. The survey was conducted at the door step of each household. If there were more families under one physical structure, they were considered as two families. Details of head of the household and how the person is related to other members of the family was collected. This helped us to accurately record the number of families in the surveyed households.

Each team was given a target of 20 households per day, assuming a total of 100 households to be screened in a day.

The survey started at 7.00 in the morning so as not to miss those who leave to work early in the morning. However, those who went to work very early in the morning and returned late in the evening could not be covered during the survey, even after multiple visits. Slum dwellers who were not available for data collection for two consecutive visits were excluded from the study.

Each person in the team had a specific assigned role. One person explained the purpose of the study to the people in the household, took consent and collected the household and family data. Two nurses set up the measurement station and recorded health parameters, and another counselled the family based on the results.

Following data collection, a referral slip linked to a nearby government or private health centre (depending on the choice of the participant) was provided for any abnormality detected through the screening.

A team of social workers, technology experts, a senior nurse and a doctor were present at the data collection site for support.

### Data safety

The application was designed in such a way that the data was encrypted, which gave controlled access to the data base using specific login IDs and passwords. The purpose of data encryption was to protect digital data confidentiality. Data encryption translated data into a code, so that only people with access to a decryption key or password can read it. One researcher based at BBH had a unique user ID and password, which allowed them to access all data to allow appropriate follow up as required. All other partners had unique user ID and password which provided access to de- identified data.

### Standard definitions, measurements and cut off used

The standard cut off is summarized in Table 2.

**Table 2:** Standard cut offs used for data collection

Standard definitions	Cut offs used
Body Mass Index [15]	
Normal	BMI values 18.5 kg/m^2^–22.9 kg/m^2^
Overweight	BMI value 23.0 kg/m^2^ to 24.9 kg/m^2^
Obesity	BMI above 25 kg/m^2^
Hypertension JNC 7 [16]	
Normal	SBP < 120 mm of Hg and DBP < 80 mm of Hg
Prehypertension	SBP 120–139 mm of Hgor DBP 80–89 mm of Hg
Stage 1 Hypertension	SBP 140–159 mm of Hgor DBP 90–99 mm of Hg
Stage 2 Hypertension	SBP ≥160 mm of Hgor DBP ≥ 100 mm of Hg
Diabetes [17]	
Diabetes	FPG > 126 mg/dL (7.0 mmol/L)or RPG > 200 mg/dL(11.1 mmol/L)
Impaired Glucose Tolerance	FPG 140 to 199 mg/ dL (7.8 to 11 mmol/L)or RPG 100 to 125 mg /dL(5.6 to 7.0 mmol/L)
Anaemia [18]	
Mild	Hb values 11.9–11.0 g/dL
Moderate	Hb values 10.9–8.0 g/dL
Severe	Hb value < 8.0 g/dL
Malnutrition indices in children [19]	Definitions
Underweight	Weight/age < − 2 standard deviations (SD) of the WHO Child Growth Standards median
Stunting	Height/age < − 2 standard deviations (SD) of the WHO Child Growth Standards median
Wasting	Weight/height < − 2 standard deviations (SD) of the WHO Child Growth Standards median

*BMI* Body mass index, *SBP* Systolic blood pressure, *DBP* Diastolic blood pressure, *FPG* Fasting plasma glucose, *RPG* Random plasma glucose, *Hb* Haemoglobin

All the instruments were calibrated by the biomedical department as per standards.

BMI: The body mass index is a physical measurement used to assess the total amount of body fat. It was calculated by dividing weight in kilograms (kg) by the square of height in metres (m^2^). BMI was calculated for all adults (18 years and above) and a person with BMI within normal range was considered as normal [15].

Hypertension: Blood pressure (BP) was recorded in the sitting position in the right arm with aneroid sphygmomanometer for all adults 30 years and above. A person who is on anti- hypertensives or a person who has elevated blood pressure was considered as hypertensive [16].

Diabetes: Diabetes screening was done using glucometer for all adults 30 years and above. A person who is on anti-diabetic medication or has elevated fasting plasma level or an elevated random plasma glucose, was considered as diabetic [17].

Anaemia: Anaemia screening was done on females 12 years and above using WHO Haemoglobin Colour Scale (HCS) [20]. A person with a hemoglobin (Hb) value less than normal was considered as anaemic [18].

Malnutrition in children: For all children less than 5 years, weight and height were measured using Salter bathroom scale and measuring tape respectively. For children less than two years, tared weighing (mother and child deduction method) was done. If a child was less than 2 years old, child’s length was measured in the lying down (recumbent) position using the infantometre which was placed on a flat, stable surface. If the child is aged 2 years or older, standing height was measured. Weight for age, height for age and weight for height and corresponding Z scores were calculated using WHO anthro software [19]. A child was considered as normal when all the nutritional parameters (weight for age, height for age, weight for height and BMI for age) measured were within normal limits as per World Health Organisation (WHO) guidelines.

### Measures to reduce bias

Selection bias was minimised by screening all the households of the selected zones rather than selecting few houses. Surveying team was trained and a pilot run was conducted using calibrated instruments. Team of experts were present during the data collection. This would have reduced interviewer bias and measurement bias. Subject bias have been reduced by orienting the community through meetings and using surveyors who were familiar to the area. Data handling bias was reduced by software application.

### Statistical analysis

The data collected was exported into Statistical Packages for Social Sciences version 16 (SPSS Inc. Released 2007. SPSS for Windows, Version 16.0. Chicago, SPSS Inc.).Descriptive statistics were used to summarize household and family parameters. The Crude death rate of the population was calculated by dividing total deaths reported in the last year by the total sample population, multiplied by 1000 [21]. The prevalence of hypertension and diabetes was expressed in percentages. The association between education level and gender was analysed using a Chi-square test. A *p*-value of < 0.05 was considered as significant.

## Results

All the houses (2143) in the six chosen zones were mapped. All the houses in five zones were completely surveyed and the last zone (Thangamalai Nagar, Cluster A) remained partial since recruitment was stopped once the desired sample size was achieved. Almost a third (29.2%) of these houses were locked at the time of survey, 11.6% of the mapped houses were shops and 84 (3.9%) households refused to participate in the study. The families in the remaining 1186 (55.3%) households were screened during the survey. Seven hundred and fifty five (63.7%) of these households belonged to the Cluster B.

### Data collected at the household and family levels

#### Living situation

The majority (63.6%) of the houses had a cement floor and a concrete roof. A small proportion of houses (7.7%) had mud floor and asbestos roof, 94.5% of those belonged to cluster B. Most houses (68%) had only one room. Different portions of the same room were used to cook, sleep and wash. Almost a third of the households (28.9%), did not have access to the public food distribution system. The majority (70.4%) had a Below Poverty Line (BPL) Card which provides access to subsidised food allowances and medication. A handful of households (4.1%) had medical insurance .

#### Health

##### Mortality

Forty households reported death of a member in the last year. The Crude death rate is 10.83/1000 population. Many deaths (35%) happened amongst the elderly (> 60 years) but a high proportion (50%) of deaths occurred in relatively younger age groups (20–59 years). The most common cause of death (30%) in all the households reported was heart attack. Other causes include road traffic accidents, tuberculosis, alcohol related deaths and other infections. Half of the people who died from a heart attack were younger than 50 years. In 12.5% of the cases, the cause of death was unknown. No pregnancy related deaths were reported.

#### Health seeking behaviour

Most people (61.2%) sought help from a private clinic when they fell ill. Almost a third (31.3%) of the households reported some kind of morbidity.

### Data collected at the individual level

#### Age and gender distribution

In the 1186 participating households, 3693 people completed the survey and were screened with the THULSI kit. Among the screened population, the majority (61.2%) were females. Infants and adolescents constituted 2.9 and 21.4% of the screened population respectively. The most common age group was between 20 and 39 years (32.1%). Elderly (60 years and above) constituted 6.1% of the screened population.

#### Literacy

There were 691 men and 1367 women above the age of 18. Almost half (40.2%) of the adult population could not read or write in any one language. Female literacy was significantly lower compared to males (42.6% vs. 54.7%; *p* = 0.009). While comparable data is not available for children, 91.9% of the children of school going age, reported to be attending school.

#### Occupation

Almost half (42.3%) of the adult population screened had an occupation. Manual labourer at a building construction site and working as a domestic maid were the most common occupations of men and women respectively. Only33.1% had a regular job (at least 3 days in a week). The average daily income was INR 304.2(USD 4.3) [22]. The average income was less in women as compared to men (USD 2.6 vs USD 5.3) [23]. Further, the unemployment rate was higher in women (72.6%) as compared to men (16.2%). Twenty four boys and six girls were found to be below the legal age for working.

#### Morbidity

The prevalence of obesity, hypertension, diabetes and anaemia is presented in Table 3.

**Table 3:** Prevalence of morbidity among screened population

Morbidity	Screened population	Classification	Number of persons	Percentage among those screened
Body mass index	Measured for 18 years and above None refused *N* = 2033	Normal	644	31.7
		Obese	805	39.6
		Overweight	290	14.3
		Underweight	294	14.5
Hypertension	Screened for 30 years and above None refused *N* = 1285	Normal Blood Pressure	748	58.2
		Pre hypertension	82	6.4
		Hypertension	456	35.5
		Newly detected	258	20.1
Diabetes	Screened for 30 years and above 23 refused *N* = 1262	Normal	907	71.9
		Impaired Glucose Tolerance	146	11.6
		Diabetes	209	16.6
		Newly detected	72	5.7
Anaemia	Screened for woman 12 years and above 25 refused *N* = 1611	Normal	469	29.1
		Mild anaemia	918	57
		Moderate anaemia	184	11.4
		Severe anaemia	40	2.5

##### Obesity

The majority (68.7%) of the obese adults were under 45 years of age and were females (76.9%). Most (80.2%) of the obese and overweight people were married.

##### Hypertension

Among the hypertensives, more than half of them were newly detected. Among those newly detected as being hypertensive, 59.7% were female and 53.1% were younger than 50 years. Another important observation was that 33.3% had stage 2 hypertension, among which 10.5% were also newly diagnosed to have diabetes in the screening.

##### Diabetes

A few (23 people) refused the test due to fear and anxiety of invasive test. Diabetes was newly diagnosed in 5.7% of the screened population. Among the newly detected, 68.1% were female and 52.8% were younger than 50 years. Among the diabetics, 15.3% had stage 2 hypertension and 58.3% were obese.

##### Anaemia

Prevalence of anaemia was 71.1% among adolescent girls (12–19 years). Among women with severe anaemia, 55% belonged to the age group below 30 years.

### Nutritional status of children under the age of 5 years

**Fig. 2:**
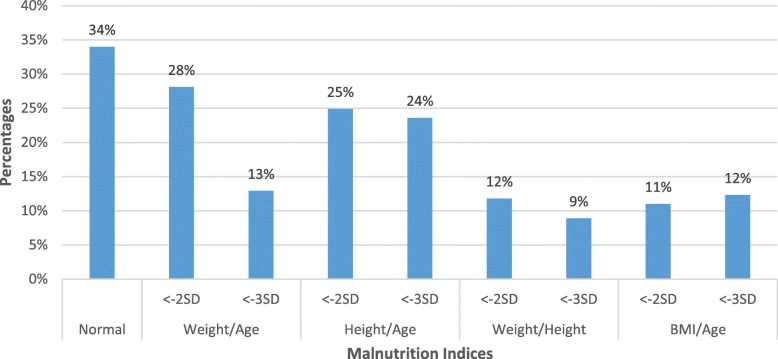
Nutritional status of children aged less than 5 years. Z scores less than 2 standard deviations were considered as malnourished. Percentages are approximated to the nearest whole number

Among those surveyed were 519 children who were younger than 5 years. However, the exact date of birth was known only for 381 of these children. Nutritional status was assessed in 318 children using standard anthropometric indicators height for age, weight for age, weight for height and BMI for age, which is depicted in Fig. 2.

The prevalence of under nutrition was 41% and of stunting was 48.5%. Of all the anthropometric parameters, stunting - which shows chronic nutritional deficiency - was most prevalent. The prevalence of malnutrition was not associated with age or gender (*p* > 0.05).

## Discussion

THULSI facilitated surveying the slum population at DJ Halli, allowing the research team to gather information about living conditions there and gather relevant health data. It enabled health professionals to go to slum households, understand their living conditions, engage in conversations about health and screen for the most common diseases. Since health seeking behaviour among slum populations is poor [6], this opportunity to conduct a health screening is of great benefit to urban slum communities. Many of those surveyed were identified as having medical problems for the first time, and it was possible to counsel and refer them to local health facilities as a result of the screening. Lack of understanding of health benefits of clinic attendance and poor attitude of the health professionals are cited as barriers for health seeking in slums [24]. THULSI enabled a deeper engagement between slum dwellers and the healthcare professionals than a regular clinic encounter. Positive engagement and supportive counselling have the potential to improve the health seeking pattern and thereby impact slum health positively over the long run.

The results of the study should be interpreted keeping in mind the limitations of the sampling strategy. Estimation of the slum size, further sub divisions of the slum and the boundaries of area zones were based on local leaders and community discussions. We did not use official government statistics for the sampling strategy because of its major discrepancy from reality. The linkages we had made with the community, our observations over the past decade of work in the slum and our collaborations with diverse experts made it possible to use best possible sampling strategy within the challenges of surveying this slum population.

The whole strategy of the survey can be summarised as empowering low skilled health teams to collect important data from a challenging slum context, using simple technological solutions with essential point of care diagnostics for priority health conditions in partnership with local community leadership and diverse experts. Difficulty of carrying paper survey forms and instruments, errors and variability in recording, unwillingness of people to come to a clinic for tests, complexity of the clinic based tests, disposal of the bio waste generated compounded with difficulty in travelling through narrow filthy lanes of slums with no address makes health surveys almost impossible. Data entry errors and going back to the field to correct wrong values is a monstrous challenge in the absence of house addresses. In a nutshell, THULSI empowered the health teams to collect health data through a simple process leveraging strength of the existing resources and reduced the effort, time and economic cost of the slum health survey.

Poverty was a stark reality in all pockets of this slum. A vast majority of the people were living in houses with only one room which was used for sleeping, cooking and bathing. It is well documented in other studies that people in slums live in the most inhumane conditions with 4–5 people in temporary dilapidated houses of just 100 ft with limited access to water and sanitation in an area which is flood prone and has limited garbage disposal [25]. These shared physical and social environments which are often referred to as ‘neighbourhood effects’, have far more health consequences than poverty alone [2].

Though half of those surveyed reported being employed, only a few had regular jobs. Many studies have also reported that a good proportion of slum population are employed in the low-paid labour intensive informal sector with poor income security. Hence, the dependency ratio is enormously high in this population due to three reasons: firstly almost half of the population belong to a dependant age group (below 19 years), secondly half of the working age group are unemployed and thirdly only very few people have regular work to sustain themselves. Therefore, most of their struggle is to find resources to pay for food and shelter, with other basic necessities, such as healthcare and education taking a back seat.

Child labour reported during the survey was less than what is anticipated in a slum settings. UNICEF reports that child labour constitutes 13.1% of the India’s workforce [26]. Though the proportion has reduced in the last decade, still two million children are working in slums and towns in India [27]. One reason may be the access to free schools in the locality which may have decreased the child labour rate. However the possibility of non-reporting of child labour due to legal implications cannot be ruled out.

Metropolitan cities promise better economic opportunities, decent and stable jobs, and better life which operates as the pull factors for urban migration [28]. However the reality is that people in urban slums get trapped in a vicious cycle of poverty due to inequity in opportunities, lack of assured daily work and difficulties in accessing health and education juxtaposed with prosperity and fast paced development.

Crude death rate is higher than the national average (10.7/1000 vs 7.1/1000) [29]. Heart attack was reported as the major killer. Unidentified diabetes and hypertension in this population can be one of the reasons for heart attacks and premature deaths. During the THULSI screening, a substantial proportion of those surveyed were diagnosed with diseases (e.g. hypertension, diabetes and anaemia) for the first time. Since access to screening and treatment are limited in slum settings, the formal health sector inevitably deals with severe and end stage complications with regard to chronic diseases [4]. It has been documented that the majority (80%) of deaths due to chronic diseases occur in low-income and middle-income countries. Further, the death rates from these potentially preventable diseases are higher in low-income and middle-income countries than in high-income countries, especially among adults aged 30–69 years [30]. Household health screening and opportunities for treatment have the potential to reduce the burden of serious health consequences in slum populations. Since health in this population is not only a function of poverty, but also of the intimately shared physical and social environments, interventions in these slums offer high returns on investment as the beneficial effects are shared across the neighbourhoods [31].

Estimated prevalence of obesity (39.6%), hypertension (35.5%) and diabetes (16.6%) in adults in the current study was concurring with the national estimates of obesity (10.1–41.5%) [32], hypertension (26.7–33.0%) [33] and diabetes(13.2–14.6%) [34]. Though the estimated overall prevalence of anaemia in this study (70.9%) is higher than national estimates (53%) [13], the proportion of moderate to severe anaemia is less in this study compared to national estimates (13.9% Vs. 16%). It was reported that under-weight among under-five children ranged from 39 to 75%, stunting from 15.4 to 74% and wasting from 10.6 to 42.3% in different parts of the country [14]. Variations can be attributed to different age groups studied (less than 3 years, 1–5 years, 0–6 months, infants) difference in criteria (WHO, Indian Academy of Paediatrics), and difference in the geographical attributes (urban, rural, tribal, slums, regions). The current study reported 40% undernutrition, 48.5% stunting and 20.7% wasting which is within the national estimates.

Lifestyle diseases such as obesity, hypertension, and diabetes were once considered as ‘diseases of affluence’ and hence least expected in poor populations. This study as well as studies conducted in slums of India [35] and other parts of the world [36] have proved these assumptions wrong. One fourth of the adult population in slums were reported to have hypertension, diabetes or obesity which is consistent with the findings of the present study [37, 38]. Migration from villages to slums has certainly altered physical activity and diet patterns, provided easy access to smoking and alcohol and exposed people to increased stress. These factors are postulated to cause increased prevalence of chronic lifestyle diseases in these populations [37].

Even in developed countries, poverty is linked with obesity [38]. Fresh fruits and vegetables are expensive compared to unhealthy food. Hence impoverished areas are oftentimes called “food deserts,” implying diminished access to fresh food [38]. The practicality of home cooked food is questionable when the food has to be cooked in a 100 ft. house, where 3–4 children are moving around with limited availability of water and ventilation. Thus, the non-financial cost of home cooking is enormously high if factors such as the difficulty of carrying water, fear of burns and injury to children, unpleasant effect of soot and smoke in the house and the agony of throwing away excess food due to non-availability of refrigeration, are considered. Hence, slum communities often resort to easily available, cheap, low nutritive junk food which may be implicated in both obesity and undernutrition in slums.

It was shocking to see high prevalence of undernutrition in children and obesity in adults in the midst of poverty and poor food security. This phenomenon is documented in studies as the double burden of malnutrition. The WHO defines double burden of malnutrition as the coexistence of undernutrition along with overweight and obesity, within individuals, households and populations, and across the life course [39]. Studies from Indonesia have documented household food insecurity as a predictor for Stunted Children and Overweight/Obese mothers (SCOWT) [40].

Anaemia among women is a well-documented public health problem among the developing countries of the world, especially in South Asia. Global estimates of anaemia average around 56%, ranging from 35 to 75% depending upon on the geographical location [41]. Our study findings are consistent with the statistics reported from the National Family Health Survey (NFHS) carried out in India, which recorded the highest prevalence of anaemia amongst the urban poor compared to their rural counterparts [42, 43]. Poor housing, overcrowding, pollution, increased exposure to infectious diseases and reproductive tract infections, coupled with physical and financial barriers to healthcare access are important causes of anaemia in this population [44]. Diminished autonomy and gender inequity in slums results in complex dynamics of early and frequent fertility cycles and reduced economic power within the household [45] which limits access to nutritional food and healthcare. These factors may be responsible for the high prevalence of anaemia in this population.

Poor nutritional indicators in children, especially stunting, indicate poor environmental conditions and long-term restriction of a child’s growth potential. The WHO has laid down ≥30%, ≥40% and ≥ 15% for underweight, stunting and wasting respectively as serious and critical values of public health importance in a population [46]. In our study, all the malnutrition indices (41, 48.4 and20.7%) for children under the age of 5 years exceeded the maximum threshold levels established by the WHO. Nutritional insult of this magnitude can impair the cognitive, social, emotional and physical development of the younger slum population and thereby trap them in poverty forever.

The study has a few limitations. The first is that those who went to work early morning and returned late could not be covered during the survey, even after several visits. The application did not allow to take the demographic details of the physically absent person in a household, during the survey. This feature necessitated the survey team to revisit the household to collect the data of the missing person. This was a feature purposively built in the application to improve the accuracy of collected data. Hence it is unknown how many persons were missed due to this reason, but we believe that they comprise a small proportion of the population. Environmental constraints like lack of flat surface (weight and height measurement), fragility of roof to hang salter scale (weight measurement) and dim light (reading haemoglobin colour strips) posed methodical challenges in estimating parameters which would have caused errors. Surveyors were trained adequately to deal with such situations were instructed to seek help from the experts who were present in the field. No repeat measures were taken if the blood sugar or blood pressure was high. This might have resulted in the overestimation of parameters. Another limitation was the difficulty to reach people, to map the slum and to find ways to follow-up on the visits. Finally, the present study focused only on the population of one slum. This may have an effect on the external validity of the study.

Following the conclusion of the study, BBH started several initiatives based on the health screening data. Community meetings were held and mobile clinics were started in the slum pockets where there was high incidence of morbidities. To tackle the problem of malnutrition, Project “Little Einstein”, that aims at improving the nutritional status of the children in slums, was initiated. Mothers meeting, nutritional counselling and supplementation, health worker and nurse home visits and capacity building programmes for grassroots nutrition workers were part of this initiative. The THULSI toolkit and application have been refined on the basis of the experiences with this survey and are now being used on a regular basis.

## Conclusions

We conducted a health survey in the challenging context of a large notified slum in South India to estimate the demographic and priority health parameters using a simple mobile screening toolkit. Poor structures, poverty, lack of decent employment, high prevalence of hypertension, diabetes, and anaemia and childhood malnutrition was reported. This will contribute evidence to slum health. The study also generates evidence for the feasibility of simple technological solution in collecting health data by reducing time, effort and economic cost of the slum health survey and generating valuable data for designing programs to improve slum health.

Slums pose a huge challenge and a unique opportunity to the existing health system. Relatively simple technological solutions like THULSI have enormous potential to improve slum health by providing opportunities to screen the slum population, and design suitable targeted health interventions.

## Additional file

Additional file 1: Design of THULSI prototype. (PDF 430 kb)

## Abbreviations

App: Application
BBH: Bangalore Baptist Hospital
BMI: Body mass index
BP: Blood pressure
BPL: Below poverty line
DBP: Diastolic blood pressure
DJ Halli: Devarjevanahalli
DM: Diabetes mellitus
EPSRC: Engineering and Physical Sciences Research Council
FPG: Fasting plasma glucose
GCRF: Global challenges research fund
Hb: Haemoglobin
HTN: Hypertension
ID: Identity document
JNC: Joint National Committee
NFHS: National Family Health Survey
RPG: Random plasma glucose
SBP: Systolic blood pressure
SCOWT: Stunted Children and Overweight/Obese mothers
SD: Standard deviation
SPSS: Statistical Packages for Social Sciences
THULSI: Toolkit for Healthy Urban Life in Slums Initiative
UN-Habitat: The United Nations Human Settlements Programme
UNICEF: The United Nations International Children’s Emergency Fund
USD: United States Dollar
WHO: World Health Organization
