# Supplementary figures and images for: Health issues in a Bangalore slum: findings from a household survey using a mobile screening toolkit in Devarajeevanahalli
Source: BMC Public Health. 2019 Apr 29;19:456. doi: 10.1186/s12889-019-6756-7 (PMC6489349; doi:10.1186/s12889-019-6756-7)

# Design for Kit A and Kit B of THULSI

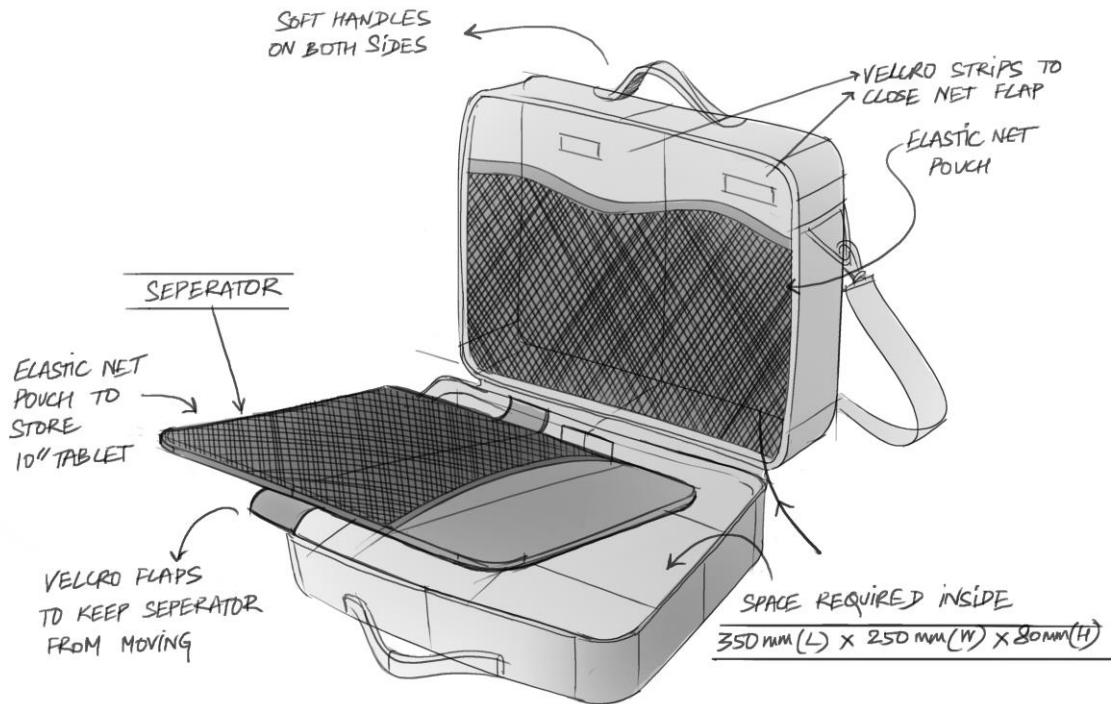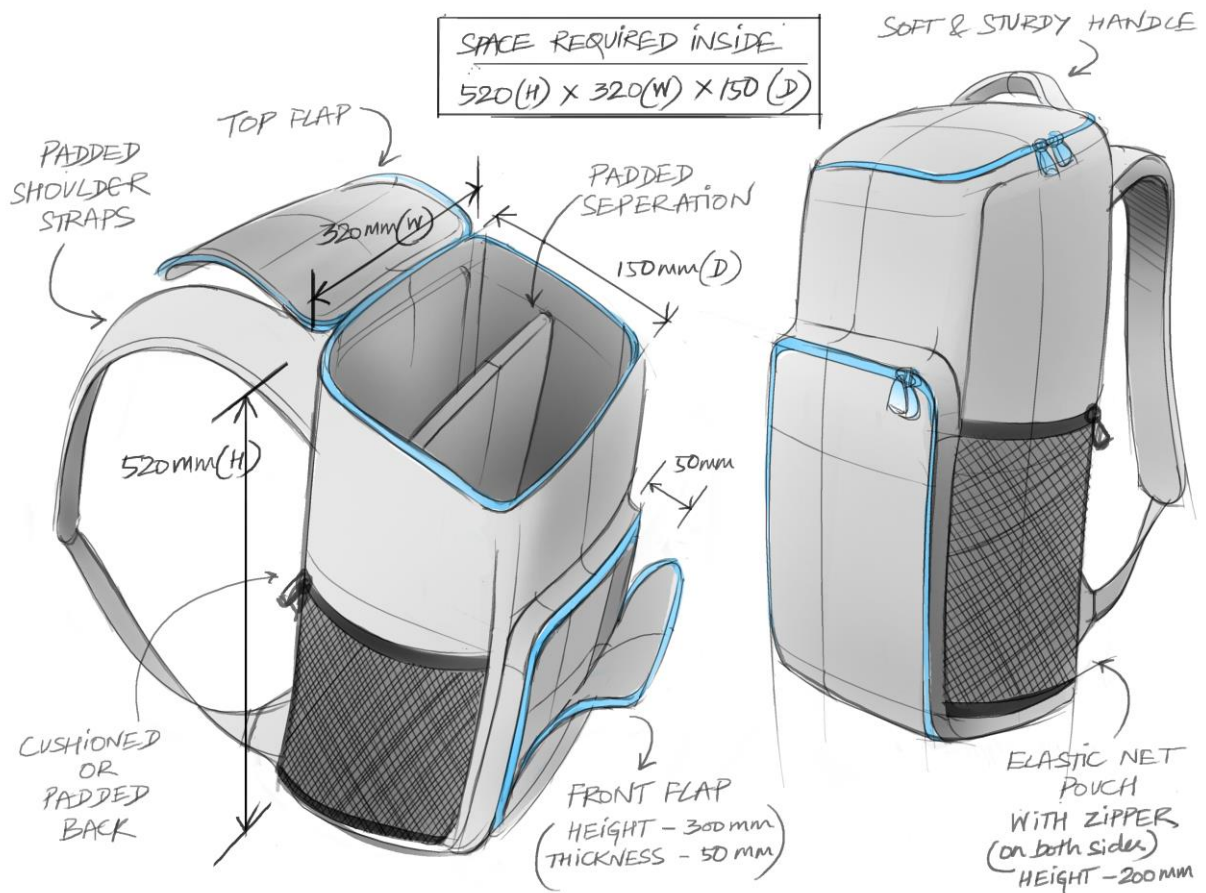

Supplement: Supplementary file 1 — Design of THULSI prototype. (PDF 430 kb) [file 12889_2019_6756_MOESM1_ESM.pdf]
